# Neuromelanin, neurotransmitter status and brainstem location determine the differential vulnerability of catecholaminergic neurons to mitochondrial DNA deletions

**DOI:** 10.1186/1756-6606-4-43

**Published:** 2011-12-21

**Authors:** Matthias Elstner, Sarina K Müller, Lars Leidolt, Christoph Laub, Lena Krieg, Falk Schlaudraff, Birgit Liss, Chris Morris, Douglass M Turnbull, Eliezer Masliah, Holger Prokisch, Thomas Klopstock, Andreas Bender

**Affiliations:** 1 Department of Neurology with Friedrich-Baur-Institute, Ludwig-Maximilians-University, 81377 Munich, Germany; 2 Integrated Center for Research and Treatment of Vertigo, Balance and Ocular Motor Disorders, Ludwig-Maximilians-University, 81377 Munich, Germany; 3 Insitute of Applied Physiology, Ulm University, 89081 Ulm, Germany; 4 Institute for Ageing and Health, Newcastle University, Newcastle upon Tyne NE4 5PL, UK; 5 Medical Toxicology Centre, Wolfson Unit of Clinical Pharmacology, Institute of Neuroscience, Newcastle University, Newcastle upon Tyne NE2 4AA, UK; 6 Institute of Ageing and Health, Newcastle University Centre for Brain Ageing and Vitality and Mitochondrial Research Group, Newcastle University, Newcastle upon Tyne NE2 4HH, UK; 7 Experimental Neuropathology, University of California in San Diego (UCSD), La Jolla, CA, 92093, USA; 8 Institute of Human Genetics, Technical University Munich, 81675 Munich, Germany; 9 Helmholtz Zentrum Munich, German Research Center for Environmental Health, 85764 Neuherberg, Germany; 10 Department of Neurology, Therapiezentrum Burgau, 89331 Burgau, Germany

**Keywords:** Parkinson disease, aging, neurodegeneration, catecholaminergic neurons, mitochondrial DNA, single neuron analysis, laser-microdissection

## Abstract

**Background:**

Deletions of the mitochondrial DNA (mtDNA) accumulate to high levels in dopaminergic neurons of the substantia nigra pars compacta (SNc) in normal aging and in patients with Parkinson's disease (PD). Human nigral neurons characteristically contain the pigment neuromelanin (NM), which is believed to alter the cellular redox-status. The impact of neuronal pigmentation, neurotransmitter status and brainstem location on the susceptibility to mtDNA damage remains unclear. We quantified mtDNA deletions (ΔmtDNA) in single pigmented and non-pigmented catecholaminergic, as well as non-catecholaminergic neurons of the human SNc, the ventral tegmental area (VTA) and the locus coeruleus (LC), using laser capture microdissection and single-cell real-time PCR.

**Results:**

In healthy aged individuals, ΔmtDNA levels were highest in pigmented catecholaminergic neurons (25.2 ± 14.9%), followed by non-pigmented catecholamergic (18.0 ± 11.2%) and non-catecholaminergic neurons (12.3 ± 12.3%; p < 0.001). Within the catecholaminergic population, ΔmtDNA levels were highest in dopaminergic neurons of the SNc (33.9 ± 21.6%) followed by dopaminergic neurons of the VTA (21.9 ± 12.3%) and noradrenergic neurons of the LC (11.1 ± 11.4%; p < 0.001). In PD patients, there was a trend to an elevated mutation load in surviving non-pigmented nigral neurons (27.13 ± 16.73) compared to age-matched controls (19.15 ± 11.06; p = 0.052), but levels where similar in pigmented nigral neurons of PD patients (41.62 ± 19.61) and controls (41.80 ± 22.62).

**Conclusions:**

Catecholaminergic brainstem neurons are differentially susceptible to mtDNA damage. Pigmented dopaminergic neurons of the SNc show the highest ΔmtDNA levels, possibly explaining the exceptional vulnerability of the nigro-striatal system in PD and aging. Although loss of pigmented noradrenergic LC neurons also is an early feature of PD pathology, mtDNA levels are not elevated in this nucleus in healthy controls. Thus, ΔmtDNA are neither an inevitable consequence of catecholamine metabolism nor a universal explanation for the regional vulnerability seen in PD.

## Background

Oxidative stress and mitochondrial dysfunction are believed to have a dominant role in mechanisms of aging and neurodegenerative disorders such as Parkinson disease (PD) [1]. The mitochondrial theory of aging proposes that production of reactive oxygen species (ROS) in mitochondria causes accumulating damage to proteins, lipids, and mitochondrial DNA (mtDNA). As a consequence, mitochondrial dysfunction and ROS production may build up in a vicious cycle that eventually results in cell death [2,3]. Damage to mtDNA is central to this theory and early studies provide evidence for the accumulation of somatic mtDNA deletions (ΔmtDNA) in aging postmitotic tissues with high energy demand, such as skeletal muscle and the brain [4,5]. Nevertheless, due to the low abundance of ΔmtDNA detected in crude tissue homogenates, their functional significance remained controversial. By combining laser-microdissection (LMD) with a quantitative real-time PCR (RT-PCR) assay, we demonstrated the age-related accumulation of clonally expanded ΔmtDNA in individual post mortem dopaminergic neurons of the substantia nigra pars compacta (SNc) [6]. Indicative of a resulting functionally relevant biochemical defect, neurons with high levels (~ 60%) ΔmtDNA had mitochondrial cytochrome-c oxidase (COX, complex IV of the mitochondrial respiratoy chain) deficiency on histochemical examination in PD patients and controls. These findings were independently confirmed using a different methodological approach [7]. Our studies into the regional distribution of ΔmtDNA further showed that dopaminergic nigral neurons have a higher propensity to accumulate ΔmtDNA than extranigral populations, e.g. in the putamen, the hippocampus or the frontal cortex [8,9]. Besides their catecholaminergic neurotransmitter status, a prominent feature of these neurons is their pigmentation, i.e. the intraneuronal accumulation of neuromelanin (NM). NM has long been considered a cellular waste product via the non-enzymatic oxidization of dopamine or other catecholamines, but some evidence points towards a regulated production that might involve alpha-synuclein [10,11]. Its contribution to neurodegenerative processes is far from understood as there is evidence for both neuroprotective and neurotoxic properties [12-14]. As a possible mechanism, it has been proposed that NM might serve to control iron homeostasis within pigmented neurons [15]. If the iron chelation ability of NM is reduced, increased levels of intra-neuronal free iron may stimulate ROS production. In PD, pigmented neurons contain less NM than in healthy brains, while the optical density of the pigment is increased [16,17]. These changes of neuronal NM content and composition may cause a loss of protective properties [18]. Indeed, we have previously shown that individual dopaminergic neurons have elevated iron levels in PD [19]. Thus, increased ROS generation, mtDNA mutations and mitochondrial dysfunction might pave the way for neurodegenerative processes in PD [20].

In this context the question arises, if location, neurotransmitter status or the presence of NM determines the vulnerability of dopaminergic nigral neurons to accumulate mtDNA damage. Herein, we investigated the association of these factors with ∆mtDNA levels in single post mortem catecholaminergic neurons that were dissected from the SNc, the ventral tegmental area (VTA) and the locus coeruleus (LC) of post mortem brains of healthy aged individuals and PD patients.

## Results

### Association of pigmentation and ΔmtDNA levels

In the adult human brain, NM is easily identifiable as a black-brown pigment by light microscopy. NM-containing neurons are distributed throughout the entire brainstem, but the largest clusters are found in the SNc, the VTA and the LC [18]. In a pilot experiment, we randomly collected pigmented neurons from the SNc of five healthy controls (80.8 ± 8.6 y) and compared ΔmtDNA levels to those seen in non-pigmented neurons within the same specimens. We found that non-pigmented neurons had a mean of 31.0 ± 25.1% ΔmtDNA, whereas pigmented neurons had a mean of 49.2% ± 18.3% ΔmtDNA (p = 0.017). This preliminary data suggested that an increased vulnerability is associated with NM-pigmentation of midbrain neurons in healthy aged controls.

**Figure 1:**
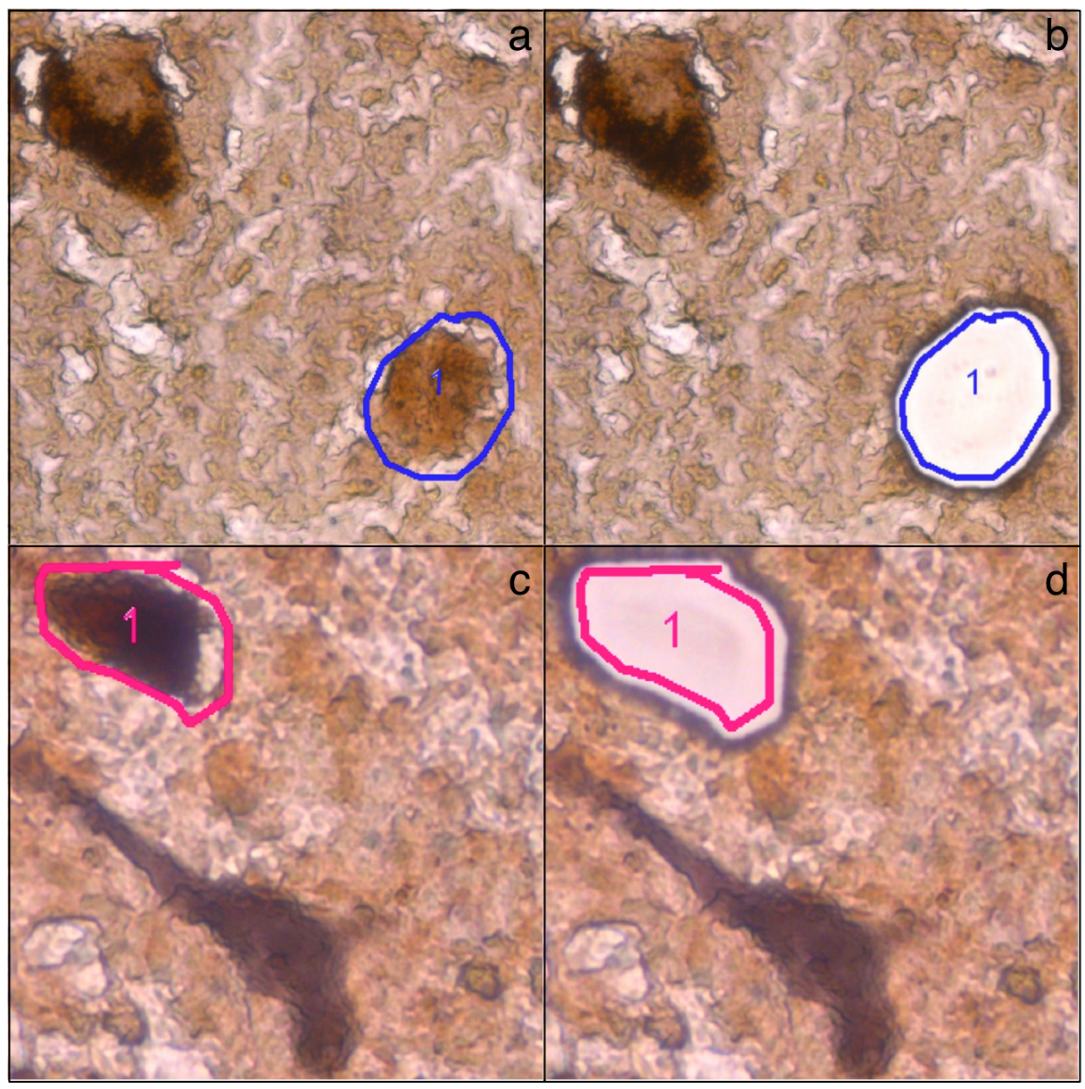
**Immunohistochemical identification of target neurons**. (a+b) Pigmented vs. non-pigmented catecholaminergic neurons. TH^+ ^neurons were identified by their brown cytosolic reaction product. Pigmented (TH^+^/NM^+^; top left) and non-pigmented (TH^+^/NM^-^; bottom right, marked for LMD) neurons were further distinguished by the visible presence or absence of NM. (a) TH^+^/NM^- ^neuron before and (b) after LMD. (c+d) Catecholaminergic vs. non-catecholaminergic neurons. NeuN^+ ^immunoreactivity results in a grey appearance of TH^- ^neurons due to a DAB/Nickel reaction (bottom neuron). (c) TH^+^/NM^+ ^neuron before and (d) after LMD.

Encouraged by these results, we next established an immunohistochemical staining protocol to facilitate a more specific and extensive analysis of catecholaminergic neurons of the SNc, the VTA and the LC. Using a successive TH/NeuN antibody labeling and DAB/HRP reaction, catecholaminergic (TH^+^) neurons were positively discriminated from non-catecholaminergic (TH^-^) neurons (e.g. GABAergic interneurons) and from glia cells. TH^+ ^immunoreactivity results in a brown cytosolic reaction product, while TH- neurons remain unstained. NeuN immunoreactivity results in a grey appearance of TH^- ^neurons due to the successive DAB/Nickel reaction. Pigmented and non-pigmented neurons were further distinguished by the visible presence or absence of NM (Figure 1). For these studies, we extended the analysis with an independent group of 19 controls (68.8 ± 19.4 y) and 14 PD specimens (75.1 ± 7.8 y).

We first asked, whether our initial data could be validated after immunhistochemical identification of catecholaminergic neurons. To this end, we analyzed ΔmtDNA levels of TH^+ ^neurons captured from all three regions (SN, VTA and LC). In a combined analysis of all regions, we confirmed a significant difference of mtDNA levels between non-pigmented (18.0% ± 11.2%) and pigmented neurons (25.2% ± 14.9%; p = 0.003). Thus, in catecholaminergic brainstem neurons, the presence of NM is associated with higher ΔmtDNA levels, independent of location and dopaminergic or noradrenergic neurotransmitter status.

### Influence of neurotransmitter status on ΔmtDNA levels

**Figure 2:**
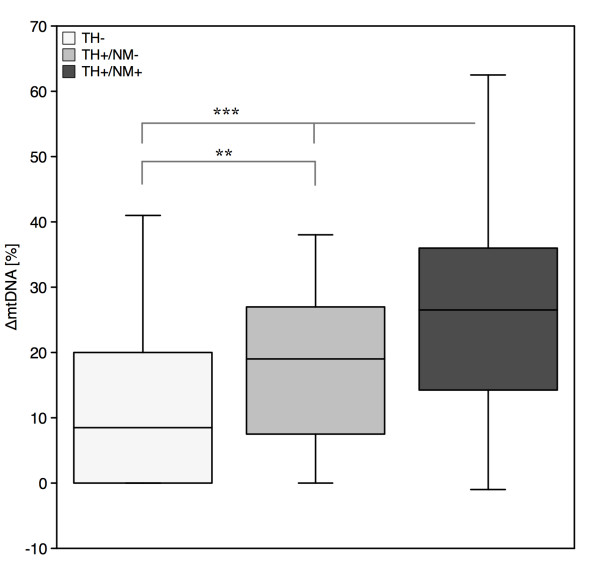
**Differential susceptibility of catecholaminergic pigmented neurons to deletions of mitochondrial DNA**. Levels of ΔmtDNA raise in the order of non-catecholaminergic (TH^-^/NeuN^+^) < catecholaminergic/non-pigmented (TH^+^/NM^-^) < catecholaminergic/pigmented neurons (TH^+^/NM^+^). Differences were significant at p = 0.005 (**; Mann-Whitney-Test) and p = 0.001 (***; Kruskal-Wallis-Test).

We next asked whether ΔmtDNA levels are generally higher in TH^+ ^(catecholaminergic) compared to TH^- ^neurons, i.e. neurons that contain neither dopamine nor noradrenaline. For this analysis, TH^-^/NeuN^+ ^neurons were additionally sampled from SN, VTA and LC. RT-PCR data showed that TH-negative midbrain neurons had low mtDNA levels of 12.3% ± 12.3. After combining this and the previous data for statistical analysis, we found a significant difference between TH^- ^neurons and pigmented as well as non-pigmented TH^+ ^neurons (Mann-Whitney-Test; p = 0.005). In these three groups, levels of mtDNA increase in the order (Kruskal-Wallis-Test; p < 0.001): non-catecholaminergic (12.3%) < catecholaminergic/non-pigmented (18.0%) < catecholaminergic/pigmented (25.2%) (Figure 2).

### Vulnerability of SNc, VTA and LC neurons

**Figure 3:**
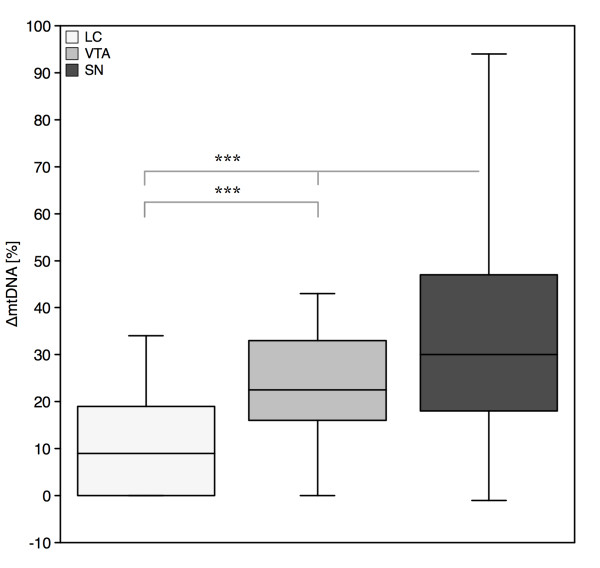
**Differential vulnerability of SNc, VTA and LC neurons to deletions of mitochondrial DNA**. Lowest levels were seen for noradrenergic neurons of the LC, followed by dopaminergic neurons of the VTA and the SNc. Differences were significant at p = 0.001 (***; Kruskal-Wallis-Test).

The SNc and VTA both predominately contain dopaminergic neurons, while the LC contains noradrenergic neurons. In the SNc and in the LC the pigment is found in approximately 95% of neurons, whereas in the VTA only about 50% neurons are pigmented [11]. We therefore asked, to what extend the location has an impact on deletion levels independent of neuronal pigmentation. Combined analysis of pigmented and non-pigmented TH^+ ^neurons showed an average of 11.1 ± 11.4% ΔmtDNA in the LC, 21.9 ± 12.3% in the VTA and 33.9 ± 21.6% in the SNc. Interregional differences were significant between all groups at p < 0.001 (Figure 3). Thus, dopaminergic neurons of the SNc are more susceptible to ΔmtDNA than those of the VTA and both are more susceptible than noradrenergic neurons of the LC.

### ΔmtDNA levels in PD nigral neurons

**Figure 4:**
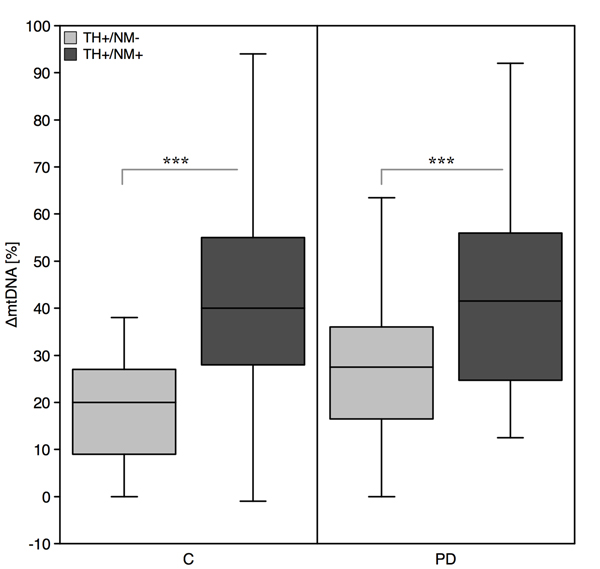
**Levels of mitochondrial DNA deletions in nigral neurons of PD and controls**. Pigmented neurons (TH^+^/NM^+^) of the SNc have considerably higher ΔmtDNA levels than non-pigmented neurons (TH^+^/NM^-^) in controls (C) and PD (*** p = 0.001). In nonpigmented neurons there was a trend to higher deletions in PD vs. controls (p = 0.052).

Lastly, we quantified ΔmtDNA levels in PD cases (n = 14; mean age 75.1 ± 7.8 years). Collection and analysis was restricted to SN dopaminergic neurons due to the paucity of suitable tissue samples. In PD, ΔmtDNA levels of pigmented neurons (41.62 ± 19.61) were again higher than those of non-pigmented neurons (27.13 ± 16.73; p = 9.6E-05), thus independently reproducing the results seen in the control group. We then compared ΔmtDNA levels in PD cases to those seen in age-matched controls (n = 19; mean age 78.7 ± 9.0 years). In non-pigmented neurons there was a trend to higher deletions in PD vs. controls (PD = 27.13 ± 16.73; controls = 19.15 ± 11.06; p = 0.052). No difference was seen for pigmented neurons in PD (41.62 ± 19.61) and controls (41.80 ± 22.62; Figure 4).

### Differential vulnerability of catecholaminergic brainstem nuclei in healthy aging

**Figure 5:**
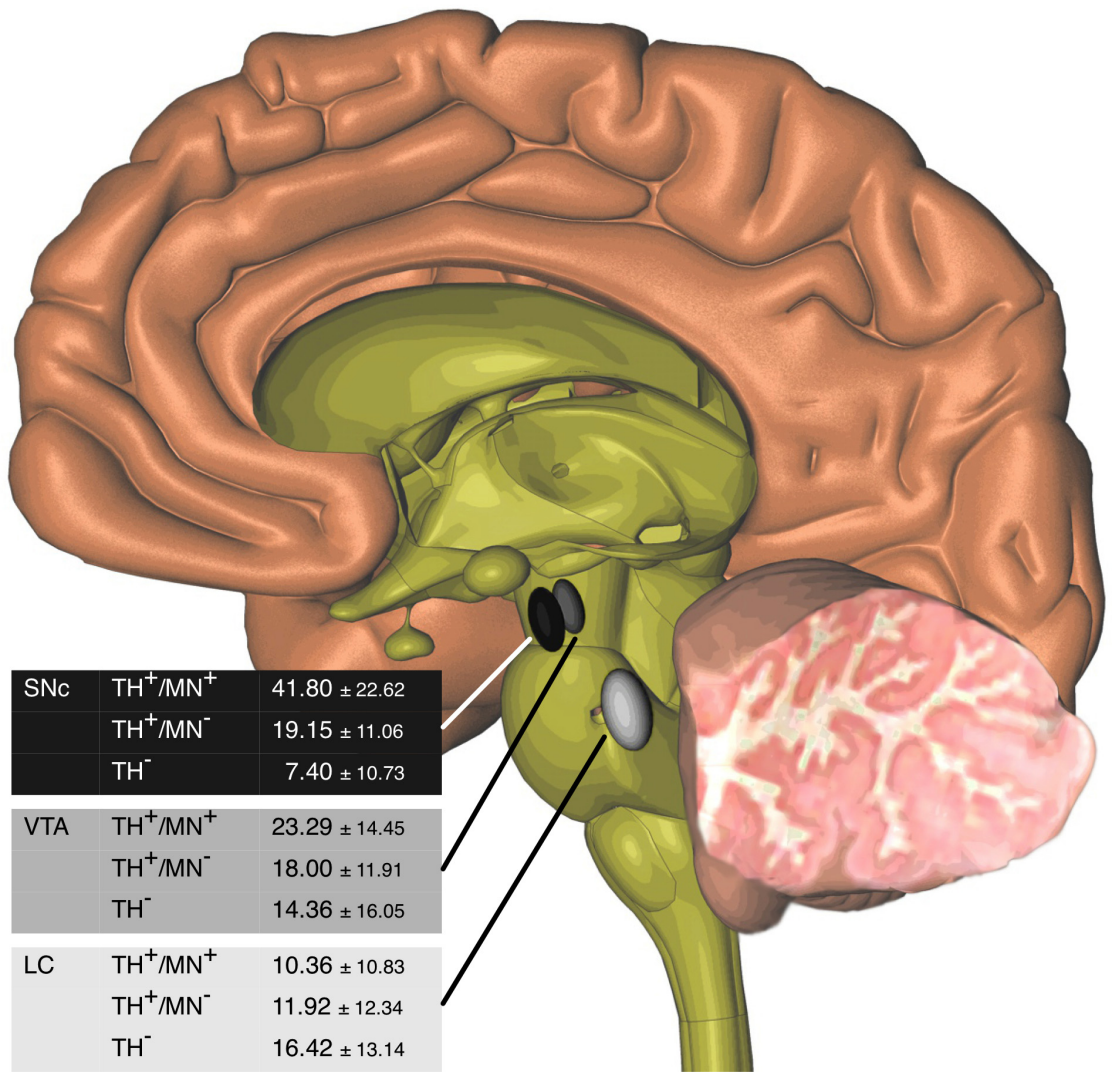
**Differential vulnerability of catecholaminergic brainstem neurons in healthy aged controls**. 3-D model of the brain showing location of brainstem nuclei and ΔmtDNA levels of pigmented (TH^+^/NM^+^) and non-pigmented (TH^+^/NM^-^) catecholaminergic neurons, as well as non-catecholaminergic (TH^-^) neurons in these nuclei. Values represent mean ± standard deviation of data collected from healthy aged controls (78.7 ± 9.0 years). Highest deletion levels are seen in pigmented neurons of the SNc (black). VTA neurons show intermediate ΔmtDNA levels (grey) and LC neurons lowest (light grey). PD pathology is deviating from this pattern, as SNc and LC show heavy degeneration whereas the VTA is relatively spared.

To generate a concise picture of ΔmtDNA levels in the aged human brain, we extracted data coming from all control individuals over 60 years of age (n = 19; mean age 78.7 ± 9.0 years). In our synopsis, individual levels of ΔmtDNA in relation to brainstem location, pigmentation and neurotransmitter status are illustrated (Figure 5). This data underlines the prominent vulnerability of pigmented nigral neurons, followed by pigmented neurons of the VTA and non-pigmented neurons of both nuclei, whereas the LC has overall low deletion levels.

## Discussion

Degeneration of pigmented dopaminergic neurons is one of the neuropathological hallmarks of PD [21,22]. In these neurons, ROS generation may be enhanced by the presence of autooxidizable dopamine, low glutathion and high iron content [23]. Additionally, recent data has revealed the reliance on Ca^2+ ^channels to maintain autonomous activity in aging dopaminergic neurons, causing sustained metabolic stress on mitochondria [24]. Decreased expression of nuclear encoded mitochondrial genes and of genes in energy-sensing pathways might further aggravate mitochondrial dysfunction [25]. The combination of these and other factors likely accelerates cellular aging processes and propagates clonal expansion of mtDNA, which are believed to arise during the repair of oxidatively damaged mtDNA [6,26,27]. COX-deficient neurons are also found in hippocampal neurons of aged individuals and Alzheimer's patients, but dopaminergic neurons of the SNc accumulate deletions to considerably higher levels [8,9]. These neurons are characterized by the age-related appearance and accumulation of NM-pigment. *In vitro *data provides an intriguing view on the potential protective and harmful properties of NM, which might change depending on the melanin species, its protein component, sulfhydryl residues and the cellular redox-state. Depletion of glutathione and upregulation of glutathione peroxidase activity in response to oxidative stress may further drive the production of neuromelanin [28]. While NM can cause apoptosis of DA neurons through an impact on mitochondrial redox state and S-glutathionylation [29], a protective role was shown in primary mesencephalic neurons under conditions of high oxidative load [30].

### Increased ΔmtDNA levels in pigmented neurons

To further elucidate the relationship of pigmentation and ROS damage in the human brain, we determined ΔmtDNA levels by RT-PCR analysis of single human post mortem brainstem neurons that were obtained from control individuals and PD patients by immunohistochemical characterization and LMD. The primary finding of this study is that ΔmtDNA levels are reproducibly elevated in neurons containing NM compared to non-pigmented neurons. To our knowledge, these data establish for the first time an association of neuronal pigmentation and mtDNA damage in human post mortem brain. Due to the limitations of a post mortem study, a causal relationship is difficult to prove or exclude. On the one hand, NM itself might cause increased ROS and mtDNA damage leading to the clonal expansion of ΔmtDNA. On the other hand, an independent pathophysiological mechanism may impose cellular stress on these neurons and cause increase in both, ΔmtDNA levels and NM content.

### Only dopaminergic neurons show elevated ΔmtDNA levels

In the literature, several lines of evidence argue against the notion that NM is a simple degradation product of catecholamine transmitter metabolism. On the contrary, NM synthesis and turnover may underlie a yet to determined enzymatic regulatory process, possibly involving alpha-synuclein [11,17,31]. While NM is found in most dopaminergic neurons of the SN and noradrenergic neurons of the LC, it does not develop in dopaminergic neurons of the olfactory bulb, some hypothalamic nuclei, nor in medullary adrenergic neurons [32]. Moreover, despite the fact that TH is the rate limiting enzyme in catecholamine synthesis there is no clear correlation between the degree of NM pigmentation and TH immunoreactivity [33,34]. We therefore asked, whether ΔmtDNA levels are generally higher in catecholaminergic than in non-catecholaminergic neurons. We found that in noradrenergic neurons of the LC, ΔmtDNA were not elevated compared to TH- neurons. In contrast to this, dopaminergic neurons of the SNc and VTA have considerably elevated ΔmtDNA levels (Figure 2) compared to other neuronal populations.

### SNc neurons are highly susceptible to ΔmtDNA - LC neurons are not

During PD disease-progression, intraneuronal pathology and neurodegeneration are seen in an 'ascending' pattern throughout the entire brain (i.e. Braak stages) [21]. Within the brainstem, catecholaminergic nuclei display a differential pathology: whereas the LC is affected early in disease and degeneration is reported to exceed that of the SNc [35], neuronal survival in the VTA is considerably higher even in severe cases [36,37]. We therefore sought to determine mtDNA mutation load in these nuclei in healthy controls. If ΔmtDNA played an integral part in the selective vulnerability in PD, a regional pattern of ΔmtDNA resembling that of Braak stages might be seen. Contrary to these expections, we found that deletion levels were considerably higher in SNc neurons than in the similarly pigmented LC neurons, (Figure 3). This raises doubts about a causative continuity of catecholamine metabolism > NM pigmentation > ROS generation > mtDNA damage. Thus, ΔmtDNA levels are not tightly associated with the differential neuronal vulnerability seen in PD (Figure 5). This finding argues against the notion that PD pathology is an acceleration of molecular events found in healthy aging, which has also been shown in morphometric studies [34]. While the agedependent loss of DA neurons is a linear process and mainly affects the dorsal part of the SNc (6,9% loss per decade), followed by the medial ventral part (5,4% per decade) and nearly sparing the ventral part (2,1% loss per decade), neurodegeneration in PD is rapidly progressive with an overall loss of DA neurons of 45% in the first decade. Furthermore, the ventral part of the SNc is the most affected part in this progress with an average loss of 95%, followed by the medial ventral part (71%) and the dorsal part (56%). It has to be stressed that - due to tissue limitations - we were not able to analyze the regional distribution of ΔmtDNA in PD samples to the extent done for controls. Therefore, we cannot rule out that ΔmtDNA levels increase to significant levels in the LC or other neuronal populations affected in PD.

### Elevated ΔmtDNA levels in SNc of PD

We further asked, whether ΔmtDNA levels reflect disease pathology in PD as was suggested by previous studies [6]. Results from PD cases independently confirmed higher ΔmtDNA levels in pigmented vs. non-pigmented neurons. Compared to controls, we found a not significant trend to higher ΔmtDNA levels in non-pigmented SNc neurons of PD brains, but this was not seen for pigmented neurons (Figure 4). The interpretation of this data is complicated by the extensive and differential neuronal loss seen in PD brains. Most severely affected neurons might have already been lost and probably only well protected and 'more resistant' neurons were left for sampling. Furthermore, within the SNc, a regional vulnerability is seen for PD: nigral neurons of the lightly pigmented ventral tier degenerate first, while the heavily pigmented of the dorsal tier are relatively preserved [16,34,38]. If pigmented neurons are mainly sampled from the pool of 'resistant' neurons located in the dorsal tier this might result in similar ΔmtDNA levels comparing PD and controls. In this study, the available midbrain samples did not allow for the discrimination of regional differences in PD, but this is planned for future studies.

Following a different line of argumentation, ΔmtDNA may simply play no relevant role in the pathogenesis of PD. Contradicting this notion, high ΔmtDNA levels are associated with a clear biochemical defect in nigral neurons and the percentage of these COX-deficient neurons is increased in the SN of PD patients [6]. Furthermore, gene defects of the mtDNA polymerase γ (POLG) result in the accumulation of ΔmtDNA and can cause parkinsonism [39]. Importantly, breakpoint analysis revealed that the types of ΔmtDNA that have clonally expanded in nigra neurons from PD patients and age-matched controls are similar to those from a patient with POLG mutations who had parkinsonism [40].

This study shows that ΔmtDNA are a common age-related phenomenon in pigmented nigral neurons, both in PD patients and healthy individuals. Thus, accumulation of ΔmtDNA clearly cannot serve as the single explanation for neurodegeneration in PD, but may precipitate dopamine neuron death in combination with other endogenous and exogenous factors. Interestingly, recent data implies that age-related accumulation of NM-pigment might induce α-synuclein expression, another important factor determining PD pathology [41].

## Conclusions

NM formation and turnover might constitute a protective system regulating neuronal redox state [11,18]. On the other hand, NM may contribute to ROS generation through release of redox-active iron under certain pathological conditions [15]. In this study, we found increased ΔmtDNA levels in pigmented midbrain neurons, which is consistent with both theories. Dopaminergic neurons have elevated levels of ΔmtDNA, supporting the role of dopamine metabolism in the generation of ROS and mtDNA damage. Importantly, in noradrenergic neurons, a causative relation of pigmentation, production of ROS and accumulation of ΔmtDNA cannot be established. Since the LC is affected early and severely in PD pathology, different factors must account for the vulnerability of catecholamingergic neurons in this nucleus.

## Methods

### Ethics statement

Frozen midbrain tissue was requested from the Newcastle Brain Tissue Resource and the German brain bank (Brain-Net^®^). Written consent was obtained with verification/assent in writing from next of kin who confirmed the wishes at time of death. All procedures were in line with the UK Human Tissue Authority guidance and approved by the Local Research Ethics Committee. The study is in accordance with the ethical standards laid down in the 1964 Declaration of Helsinki.

### Patients and controls

Control individuals had no prior history of neurological disease (n = 24, mean age 75.1 ± 7.8 years, post mortem interval (PMI) 24.0 ± 9.0 hours). Patients had a clinical and neuropathological diagnosis of PD (n = 14, mean age 71.3 ± 18.2 years, PMI 32.1 ± 18.7 hours). Neither age (p = 0.24) nor PMI (p = 0.1) were significantly different between groups. Neuropathological examination had demonstrated the presence of Lewy body pathology in the substantia nigra with typical pathological features, including moderate to severe neuronal loss and gliosis. Synuclein immunohistochemistry or ubiquitin immunohistochemistry was used to confirm findings from H&E stained sections and cases were graded according to published criteria for Lewy body disorders (LBD)[21,42].

### Histology and immunohistochemistry

Unfixed human midbrains stored at -80°C were used for analysis. 20 μm sections were cut and mounted onto Leica 2 μm PEN membrane slides (Leica Microsystems, Wetzlar, Germany) prior to staining and microdissection. For pilot experiments (n = 5 controls), sections were stained with cresyl violet (Merck, Darmstadt, Germany) and dehydrated in an ethanol series. For most experiments, a successive Tyrosin-Hydroxylase (TH) and Neuronal Nuclei (NeuN) double-staining was applied. In detail, frozen sections were fixed in ice-chilled acetone for 7 minutes and air-dried for 10 minutes at room temperature (RT). All solutions were prepared in phosphate buffered saline 1% Triton X-100 (PBST) and all incubations were performed at RT. Sections were washed twice in PBST and blocked with 5% normal goat serum (NGS) for 30 min. An anti-mouse poly horseradish peroxidase (HRP) kit was used for detection of primary antibodies according to the manufacturers protocol (Millipore, Billerica, MA, USA). Primary rabbit anti-TH antibody (abcam, Cambridge, UK) was applied at 1:300 in PBST with 5% NGS for 60 min and staining was developed with the DAB Chromogen-Buffer provided with the kit for 5 min. Sections were washed in tap water followed by blocking and incubation with 1:400 mouse IgG1 anti-NeuN (Millipore, Billerica, MA, USA) for 45 min. For successive NeuN detection, nickel chloride was added to the DAB substrate solution and developed for 10 min. The addition of nickel chloride produces a dark grey appearance of NeuN^+ ^neurons, thereby enabling distinction from the light brown of DAB-mediated TH^+ ^immunolabelling. Finally, sections were washed, dehydrated in 100% ethanol and air-dried for 30 min. Membrane sections were used for LMD immediately or frozen at -20°C for later use.

### Quantification of ΔmtDNA

UV-Laser-microdissection was performed on a Leica LMD6000 microscope (Leica, Wetzlar, Germany). Single neurons were collected into separate reaction tubes and DNA was extracted with the DNA Micro Kit (Qiagen, Düsseldorf, Germany), according to the manufacturer's protocol. Quantification of ΔmtDNA levels was based on the RT-PCR method previously described, using relative quantification of the mitochondrial ND1 and ND4 genes by means of the delta-delta-CT-method [6]. Differing from the original method, we further optimized the RT-PCR assay to be run as a duplex experiment with quantification of ND1 and ND4 genes within the same reaction. Using this protocol, there is high correlation with deletion quantification by southern blot and by the original method [43]. The following primers (MWG Biotech, Ebersberg, Germany) and TaqMan probes (Life Technologies, Carlsbad, CA, USA) were used: ND1 (forward primer nt 3485-3504, reverse primer nt 3553-3532; VIC-labeled probe nt 3506-3529) and ND4 (forward primer nt 12087-12109, reverse primer nt 12170-12140, FAM-labeled probe nt 12111-12138). Final concentrations were 900nM for primers and 250nM for probes. The Taqman Universal PCR Mastermix (Life Technologies, Carlsbad, CA, USA) was used for the assay in a 25 μl reaction mix per sample. Experiments were performed on an Applied Biosystems StepOnePlus^TM^ system (Life Technologies, Carlsbad, CA, USA). Standard cycling conditions were used as follows: activation for 2 minutes @ 50°C followed by 10 minutes @ 95°C; PCR (40 cycles) for 15 seconds @ 95°C followed by 1 minute at 60°C. Samples were analyzed in triplicates and the resulting mean values were used for statistical analysis.

### Statistical analysis and graphic design

All statistical analyses were performed with SPSS 18.0 for Mac (PASW Statistics, IBM). A total of 383 neurons were captured and mtDNA deletion levels were analyzed for every single neuron. Data of all neurons was available for analysis. Since the mtDNA deletion values did not have a normal distribution (Kolmogorov-Smirnov-Test p = 0.007), we performed non-parametric tests for all statistical procedures (Mann-Whitney-Test and Kruskal-Wallis one-way analysis of variance). Values are expressed as mean ± standard deviation. Box plots show median, lower and upper quartile. Whiskers are extended to extreme data points. Figure 5 was generated using Cinema 4D (Maxon, Friedrichsdorf, Germany).

## List of abbreviations

mtDNA: mitochondrial DNA; SNc: substantia nigra pars compacta; VTA: ventral tegmental area; LC: locus coeruleus; PD: Parkinson's disease; NM: neuromelanin; mtDNA: mtDNA deletions; ROS: reactive oxygen species; LMD: laser-microdissection; TH: tyrosine hydroxylase; COX: cytochrome-c oxidase; PMI: post mortem interval.

## Competing interests

The authors declare that they have no competing interests.

## Authors' contributions

AB, TK and ME designed the study. HP, EM, DMT aided with design details and contributed essential interpretations of findings. CM, BL and FS contributed, characterized and prepared tissue samples for the experiments. SKM, LL, CL and LK performed histochemistry and LMD. ME directly supervised experiments and wrote the paper with assistance of all authors, who have read and approved the final manuscript.
